# Calendar

**DOI:** 10.1111/iwj.13954

**Published:** 2022-09-21

**NORTH AMERICA**

**29 September – 1 October, 2022**

***Society of Vascular Medicine (SVM) 33rd Annual Scientific Sessions***

**Grand Hyatt Denver, Denver, Colorado USA**

www.woundsource.com/resource/svm-33rd-annual-scientific-sessions

**14‐16 October 2022**

***Symposium on Advanced Wound Care Fall 2022 (SAWC Fall 2022)***

**Las Vegas, NV. USA**

www.sawcfall.com

**14‐16 October 2022**

***Wounds Canada***

Sheraton Centre Hotel, Toronto Canada

www.woundscanada2022.ca

**13‐16 October 2022**

***American Vein and Lymphatic Society Annual Congress***

**New Orleans, Louisiana, USA**

www.myavls.org/annual-congress-2022

**10‐12 November 2022**

***Association for the Advancement of Wound Care***

**Salt Lake City, Utah USA**

www.aawconline.org/2022-annual-conference

**EUROPE**

**4 October, 2022**

***International Conference on Wound Care Management (ICWCM)***

**Baku, Azerbaijan**

www.conferenceindex.org/event/international-conference-on-wound-care-management-icwcm-2022-october-baku-az

**28‐29 October 2022**

***International Conference on Biomaterials in Drug Delivery and Wound Care ICBDDWC***

**Lisbon, Portugal**

www.waset.org/biomaterials-in-drug-delivery-and-wound-care-conference-in-october-2022-in-lisbon

**8‐9 November 2022**

***International Conference on Textile Materials for Wound Care ICTMWC***

**Istanbul, Turkey**

www.waset.org/textile-materials-for-wound-care-conference-in-november-2022-in-istanbul

**18‐19 November 2022**

***International Conference on Smart Textiles for Wound Care ICSTWC***

**London, United Kingdom**

www.waset.org/smart-textiles-for-wound-care-conference-in-november-2022-in-london

**24‐27 November 2022**

***16th National and 4th International Wound Congress***

**Antalya, Turkey**

www.yarakongresi2022.org/en/home-fourteen-english

**7 December, 2022**

***Israel Wounds Ostomy and Incontinence Association (IWOIA)***

**Tel Aviv, Israel**

www.facebook.com/groups/1618684384988994

**INTERNATIONAL**

**5‐6 December 2022**

***International Conference on Wound Care, Tissue Repair and Regenerative Medicine***

**Dubai, United Arab Emirates**

www.woundcare.alliedacademies.com

